# Reversible conduction failure in acute inflammatory demyelinating polyneuropathy

**DOI:** 10.1038/s41598-022-19547-0

**Published:** 2022-11-03

**Authors:** Sooyoung Kim, Eun Kyoung Lee, Eunhee Sohn

**Affiliations:** 1 Department of Neurology, Chungnam National University Hospital, Daejeon, Republic of Korea; 2 Department of Neurology, Chungnam National University Sejong Hospital, Sejong, Republic of Korea

**Keywords:** Medical research, Neurology

## Abstract

Reversible conduction failure (RCF) has been documented in acute motor axonal neuropathy (AMAN) and is considered a sign of nodopathy. Several reports of RCF in acute inflammatory demyelinating polyneuropathy (AIDP) have suggested that it could be a manifestation of nodopathy. We conducted this study to determine the frequency of RCF in AMAN and AIDP and to compare the clinical features between the two groups with or without RCF. RCF was observed in 38.9% and 18.5% patients in the AMAN and AIDP groups in our study, respectively. AIDP patients with anti-ganglioside antibodies represented 29.4% of the cohort. The clinical features of AIDP with RCF were more similar to those of AMAN with RCF than to those of typical AIDP. However, there were no significant differences in the frequency of anti-ganglioside antibody status between the groups. AIDP with RCF may be a manifestation of nodopathy. The current dichotomous electrodiagnostic criteria, classifying demyelinating and axonal neuropathy, are insufficient to define nodopathy. Further studies are required to revise the electrodiagnostic criteria for Guillain–Barré syndrome.

## Introduction

Guillain**–**Barré syndrome (GBS) subtypes can be classified according to clinical symptoms and electrodiagnostic studies. Acute inflammatory demyelinating polyneuropathy (AIDP) and acute motor axonal neuropathy (AMAN) are typical subtypes of GBS classified based on the results of electrodiagnostic studies. AMAN is diagnosed in patients who show no evidence of demyelination in electrodiagnostic studies and axonal degeneration, such as decreased distal compound muscle action potential (CMAP) amplitudes or reduced proximal CMAP to distal CMAP amplitude ratio^1^. In IgG anti-GM1 antibody-associated AMAN, conduction failure of motor nerves can be observed, which may rapidly resolve with restoration of conduction velocity and CMAP amplitudes without evidence of temporal dispersion^2–4^. It represents the pathomechanism of reversible conduction failure (RCF). RCF in AMAN has been considered a sign of nodopathy and has a favorable prognosis. Several reports have suggested that RCF in AIDP, which does not indicate consistent demyelination/remyelination, has been observed and could be a manifestation of nodopathy. However, there currently remain no precise electrodiagnostic criteria for classifying AIDP with RCF.

We conducted this study to assess the prevalence of RCF in AMAN and AIDP and to compare the clinical features and prognosis between two groups according to the presence of RCF.

## Methods

### Ethics

This study was approved by the institutional review board of Chungnam National University Hospital (approval number: 2019-09-055-002). The requirement to obtain patient consent was waived for this retrospective study by the Ehics Committee of the Chungnam National University Hospital. All the procedures were performed in accordance with the principles of the Declaration of Helsinki.

### Subjects

**Figure 1:**
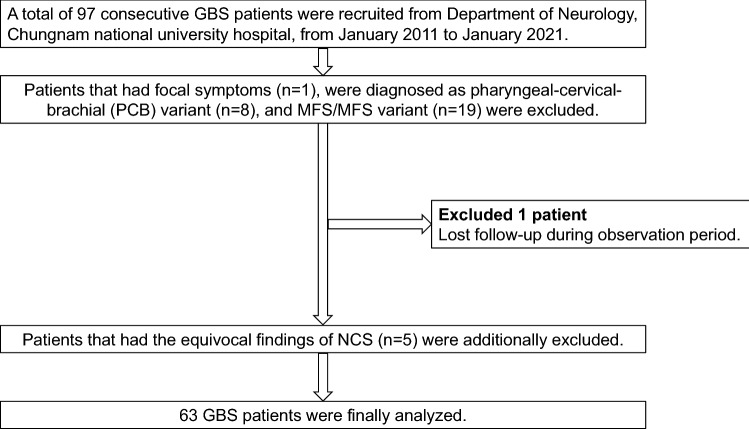
Flowchart depicting subject enrollment procedures. Ninety-seven patients with Guillain–Barré syndrome (GBS) who visited Chungnam national university hospital between January 2011 and January 2021 were initially enrolled. We included patients who had generalized weakness, which was treated with intravenous immunoglobulin, and performed nerve conduction studies (NCSs) at least twice within a month from the onset. Patients who had focal symptoms such as pharyngeal-cervical-brachial variants or were diagnosed with Miller-Fisher syndrome (MFS) and MFS variant were excluded from this study (n = 28). Of the 28 excluded patients, 8 were classified into pharyngeal-cervical-brachial variant of GBS group, 1 had focal symptom such as wrist drop, and 19 were classified into MFS and MFS variant group. One of the enrolled patients was lost to follow-up over the course of the observation period. We retrospectively reviewed the remaining 68 patients. Five of the remaining patients had equivocal findings of NCS that did not fit acute inflammatory demyelinating polyneuropathy or acute motor axonal neuropathy; therefore, these five patients were excluded. Finally, we analyzed a total of 63 patients in this study.

A total of ninety-seven patients with GBS who visited Chungnam National University Hospital between January 2011 and January 2021 were initially enrolled. We included patients who suffered from generalized weakness that were treated with intravenous immunoglobulin (IVIg) and who underwent nerve conduction studies (NCSs) at least twice within a month from the onset. Patients with focal symptoms such as pharyngeal-cervical-brachial variants or those diagnosed with Miller-Fisher syndrome (MFS) and MFS variants were excluded from this study (n = 28). Of these patients, 8 were classified as having the pharyngeal-cervical-brachial variant of GBS, 1 had focal symptoms such as wrist drop, and 19 were classified into the MFS and MFS variants. One of the enrolled patients was lost to follow-up over the course of the observation period. The remaining 68 patients were retrospectively reviewed. Five of the remaining patients had equivocal NCS findings that did not fit the AIDP or AMAN group. Therefore, these five patients were excluded. Finally, 63 patients were analyzed in this study (Fig. 1). The 63 patients were classified into the following categories: AMAN with RCF (AMAN-RCF), AMAN without RCF (AMAN-nRCF), AIDP with RCF (AIDP-RCF), and AIDP without RCF (AIDP-nRCF).

### Assessment of clinical features and electrodiagnostic findings

Demographic and clinical data, including age at onset, sex, preceding event prior to the onset, protein levels in cerebrospinal fluid (CSF) analysis (mg/dL, considered abnormal if > 45 mg/dL), presence of anti-ganglioside antibodies (anti-GM1 IgM & IgG, GD1a IgM & IgG, GD1b IgM & IgG, GQ1b IgM & IgG), period from onset to nadir, and period from onset to IVIg administration, were collected. Preceding event prior to onset was classified as upper respiratory infection, diarrhea, none, or both.

The NCS was carried out using a CareFusion Nicolet electromyography machine. Motor NCS was performed on the median, ulnar, fibular, and tibial nerves using percutaneous supramaximal nerve stimulation. An orthodromic sensory NCS was performed on the median, ulnar, and sural nerves. Patients were categorized as having AIDP or AMAN based on the Uncini’s criteria^5^. We assessed the serial NCS of all patients at least twice within a month from onset. AIDP was diagnosed in patients who had electrodiagnostic features of demyelination in at least two nerves, such as prolonged distal latency > 130% of the upper limit of normal (ULN), excluding the nerves in common entrapment sites, motor velocity slowing < 70% of the lower limit of normal (LLN), prolonged F-wave latency > 120% of the ULN, distal CMAP duration > 120% of the ULN, or proximal CMAP to distal CMAP duration ratio > 130%. AMAN was diagnosed in patients who demonstrated no evidence of demyelination at all serial NCS and had decreased distal CMAP amplitudes below 80% of the LLN or proximal CMAP to distal CMAP amplitude ratio < 0.7 in at least two nerves, excluding the tibial nerve and nerves in common entrapment sites, or absence of isolated F-wave at first NCS. In the second NCS, evidence of axonal degeneration or RCF in the two nerves should be observed. RCF in AMAN is defined as distal CMAP amplitude > 150% of that in the first NCS, without evidence of increased distal CMAP duration (≤ 120%), or a proximal CMAP to distal CMAP amplitude ratio increase of 0.2 or more compared to the first NCS, without temporal dispersion or improvement of isolated F-wave absence without delayed latencies, in at least two nerves. The definition of RCF in AIDP has not been established in current electrodiagnostic criteria. Therefore, we applied the aforementioned definition of RCF in AMAN to classify RCF in AIDP.

### Disability assessment

The Hughes Functional Grading Scale (HFGS) was used to evaluate disability in this study. HFGS reflects the severity of GBS and ranges from 0 to 6 as follows: 6, death; 5, need mechanical ventilation; 4, bedbound; 3, walk with aid; 2, walk without aid; 1, run with minor deficit; 0, normal^6^. We evaluated the HFGS of the enrolled patients at nadir, 1 and 6 months after onset. Patients with HFGS equal to or more than 3 points after 1 month from the onset were defined as high severity.

### Statistical analyses

Clinical features and prognosis of enrolled patients according to each subtype were analyzed using the chi-square test, one-way analysis of variance (ANOVA), and post-hoc multiple comparison (Bonferroni method). Clinical features and prognosis of enrolled patients classified by the presence of RCF were analyzed using the chi-square test and Mann–Whitney U test. Statistical analyses were carried out using the IBM Statistical Package for the Social Sciences (software version 26, New York, USA). *p*-value < 0.05 was considered statistically significant.

## Results

### Demographic and clinical features of the 63 enrolled patients

A total of 41 men and 22 women were enrolled in this study. The mean onset age of all patients was 58.0 ± 14.0 years. Among the 63 patients, 36 were diagnosed with AMAN (57.1%) and 27 were diagnosed with AIDP (42.9%). RCF was observed in 14 patients (38.9%) with AMAN and 5 patients (18.5%) with AIDP. In total, 17 out of 54 patients (31.5%) were positive for anti-ganglioside antibody, and the most common type of anti-ganglioside antibody was anti-GM1 antibody (64.7%). The mean period from disease onset to nadir was 6.7 ± 2.9 days and that from onset to IVIg administration was 5.9 ± 8.2 days. All patients underwent NCS at least twice from the onset. The mean period from the onset to the date of first NCS was 5.4 ± 3.6 days and that to the date of second NCS was 18.8 ± 18.4 days. Of the 63 patients, 46 underwent a third NCS, and the mean period from the onset to the date of the third NCS was 72.3 ± 47.8 days. HFGS at nadir, 1 month, and 6 months after onset were 3.4 ± 1.1, 2.5 ± 1.3, and 1.2 ± 1.4, respectively. At 1 month after the onset, 28 patients (44.4%) were found to exhibit high severity. The details of the demographic and clinical features of the 63 enrolled patients are presented in Table 1.

**Table 1:** Demographic and clinical features of the 63 patients enrolled in the study.

n = 63	
Onset age (years)	58.0 ± 14.0
Sex, male (n, %)	41 (65.1)
**Preceding event (n, %)**	
None of them	13 (20.6)
Upper respiratory infection	18 (28.6)
Diarrhea	29 (46.0)
Both of them	2 (3.2)
**Subtypes of Guillain–Barré syndrome (n, %)**	
AMAN-RCF	14 (22.2)
AMAN-nRCF	22 (34.9)
AIDP-RCF	5 (7.9)
AIDP-nRCF	22 (34.9)
Presence of RCF (n, %)	19 (30.2)
CSF protein levels (mg/dL)	62.6 ± 44.4
**Presence of anti-ganglioside antibody (n, %)**	17/54 (31.5)
Anti GM1 antibody	11/17 (64.7)
Anti GD1a antibody	2/17 (11.8)
Anti GD1b antibody	1/17 (5.9)
Anti GM1 + GD1b antibody	3/17 (17.6)
Onset to nadir (day)	6.7 ± 2.9
Onset to IVIg administration (day)	5.9 ± 8.2
Onset to 1st nerve conduction study (day)	5.4 ± 3.6
Onset to 2nd nerve conduction study (day)	18.8 ± 18.4
Onset to 3rd nerve conduction study (day), (n = 46)	72.3 ± 47.8
HFGS at nadir	3.4 ± 1.1
HFGS at 1 month after the onset	2.5 ± 1.3
HFGS at 6 months after the onset	1.2 ± 1.4
High severity (≥ 3 of HFGS) at 1 month after the onset (n, %)	28 (44.4)

AMAN, acute motor axonal neuropathy; AIDP, acute inflammatory demyelinating polyneuropathy; RCF, reversible conduction failure; AMAN-RCF, AMAN with RCF; AMAN-nRCF, AMAN without RCF; AIDP-RCF, AIDP with RCF; AIDP-nRCF, AIDP without RCF; CSF, cerebrospinal fluid; IVIg, intravenous immunoglobulin; HFGS, Hughes Functional Grading Scale.

### Clinical features and prognosis of enrolled patients according to each subtype

The age at onset of AIDP-RCF was significantly lower than that of AIDP-nRCF (AIDP-RCF, 47.6 ± 14.7 years; AIDP-nRCF, 65.0 ± 14.4 years; *p*-value = 0.01) but similar to that of AMAN-RCF (50.7 ± 13.5 years). Diarrhea as preceding event was more frequent in the AIDP-RCF, AMAN-RCF, and AMAN-nRCF groups than in AIDP-nRCF group (*p*-value = 0.002). HFGS at 1 and 6 months after the onset was significantly lower in AIDP-RCF than in AMAN-nRCF group (AIDP-RCF, 1.8 ± 0.4 at 1 month, 0.2 ± 0.4 at 6 months; AMAN-nRCF, 3.2 ± 1.3 at 1 month, 2.4 ± 1.5 at 6 months; *p*-value = 0.000 at 1 month, 0.000 at 6 months) and was similar to that in the AMAN-RCF group (1.4 ± 0.8 at 1 month, 0.3 ± 0.6 at 6 months). The frequency of high severity 1 month after onset was significantly higher in AMAN/AIDP group without RCF than in AIDP/AMAN group with RCF (*p*-value = 0.002). Nevertheless, there were no significant differences in the frequency of anti-ganglioside antibody status between the groups. The clinical features and prognoses of the enrolled patients according to each subtype are presented in Table 2.

**Table 2:** Clinical features and prognoses of enrolled patients according to each subtype.

	AMAN-RCF (n = 14)	AMAN-nRCF (n = 22)	AIDP-RCF (n = 5)	AIDP-nRCF (n = 22)	*p*-value
Onset age (years)	50.7 ± 13.5	58.1 ± 10.5	47.6 ± 14.7	65.0 ± 14.4	0.01*
Sex, male (n, %)	11 (78.6)	16 (72.7)	1 (20.0)	13 (59.1)	0.09
**Preceding event (n, %)**					0.002*
None of them	4 (28.6)	2 (9.1)	2 (40.0)	6 (27.3)	
URI	3 (21.4)	2 (9.1)	0 (0.0)	13 (59.1)	
Diarrhea	7 (50.0)	16 (72.7)	3 (60.0)	3 (13.6)	
Both of them	0 (0.0)	2 (9.1)	0 (0.0)	0 (0.0)	
CSF protein levels (mg/dL)	54.5 ± 35.8	63.0 ± 56.8	28.0 ± 11.7	75.9 ± 35.1	0.14
Presence of anti-ganglioside antibody (n, %)	4/12 (33.3)	8/21 (38.1)	2/5 (40.0)	3/16 (18.8)	0.61
Onset to IVIg administration (day)	7.7 ± 5.1	6.2 ± 13.0	3.8 ± 4.1	4.9 ± 2.8	0.73
HFGS at nadir	2.6 ± 1.0	3.9 ± 0.7	3.0 ± 1.0	3.6 ± 1.4	0.01*
HFGS at 1 month after the onset	1.4 ± 0.8	3.2 ± 1.3	1.8 ± 0.4	2.5 ± 1.3	0.000*
HFGS at 6 months after the onset	0.3 ± 0.6	2.4 ± 1.5	0.2 ± 0.4	0.9 ± 1.0	0.000*
High severity at 1 month after the onset (n, %)	2 (14.3)	15 (68.2)	0 (0.0)	11 (50.0)	0.002*

AMAN, acute motor axonal neuropathy; AIDP, acute inflammatory demyelinating polyneuropathy; RCF, reversible conduction failure; AMAN-RCF, AMAN with RCF; AMAN-nRCF, AMAN without RCF; AIDP-RCF, AIDP with RCF; AIDP-nRCF, AIDP without RCF; URI, upper respiratory infection; CSF, cerebrospinal fluid; IVIg, intravenous immunoglobulin; HFGS, Hughes Functional Grading Scale. * indicates p-value < 0.05.

Alongside the analysis of patients according to each subtype, we analyzed the clinical features and prognosis of the enrolled patients classified by the presence of RCF (Table 3). Patients with RCF had a significantly more favorable prognosis than patients without RCF (*p*-value = 0.001, HFGS at nadir; 0.000, 1 month after the onset; 0.000, 6 months after the onset; 0.000, high severity at 1 month after the onset).

**Table 3:** Clinical features and prognoses of enrolled patients classified by presence of reversible conduction failure.

	With RCF (n = 19)	Without RCF (n = 44)	*p*-value
Onset age (years)	49.9 ± 13.5	61.6 ± 12.9	0.002*
Sex, male (n, %)	12 (63.2)	29 (65.9)	1.00
**Preceding event (n, %)**			0.25
None of them	6 (31.6)	8 (18.2)	
URI	3 (15.8)	15 (34.1)	
Diarrhea	10 (52.6)	19 (43.2)	
Both of them	0 (0.0)	2 (4.5)	
CSF protein levels (mg/dL)	47.5 ± 33.2	69.3 ± 47.4	0.08
Presence of anti-ganglioside antibody (n, %)	6/17 (35.3)	11/37 (29.7)	0.76
Onset to IVIg administration (day)	6.7 ± 5.0	5.6 ± 9.3	0.62
HFGS at nadir	2.7 ± 1.0	3.8 ± 1.1	0.001*
HFGS at 1 month after the onset	1.5 ± 0.7	2.9 ± 1.3	0.000*
HFGS at 6 months after the onset	0.3 ± 0.6	1.6 ± 1.5	0.000*
High severity at 1 month after the onset (n, %)	2 (10.5)	26 (59.1)	0.000*

RCF, reversible conduction failure; URI, upper respiratory infection; CSF, cerebrospinal fluid; IVIg, intravenous immunoglobulin; HFGS, Hughes Functional Grading Scale. * indicates p-value < 0.05.

### Illustrative case of AIDP-RCF

**Figure 2:**
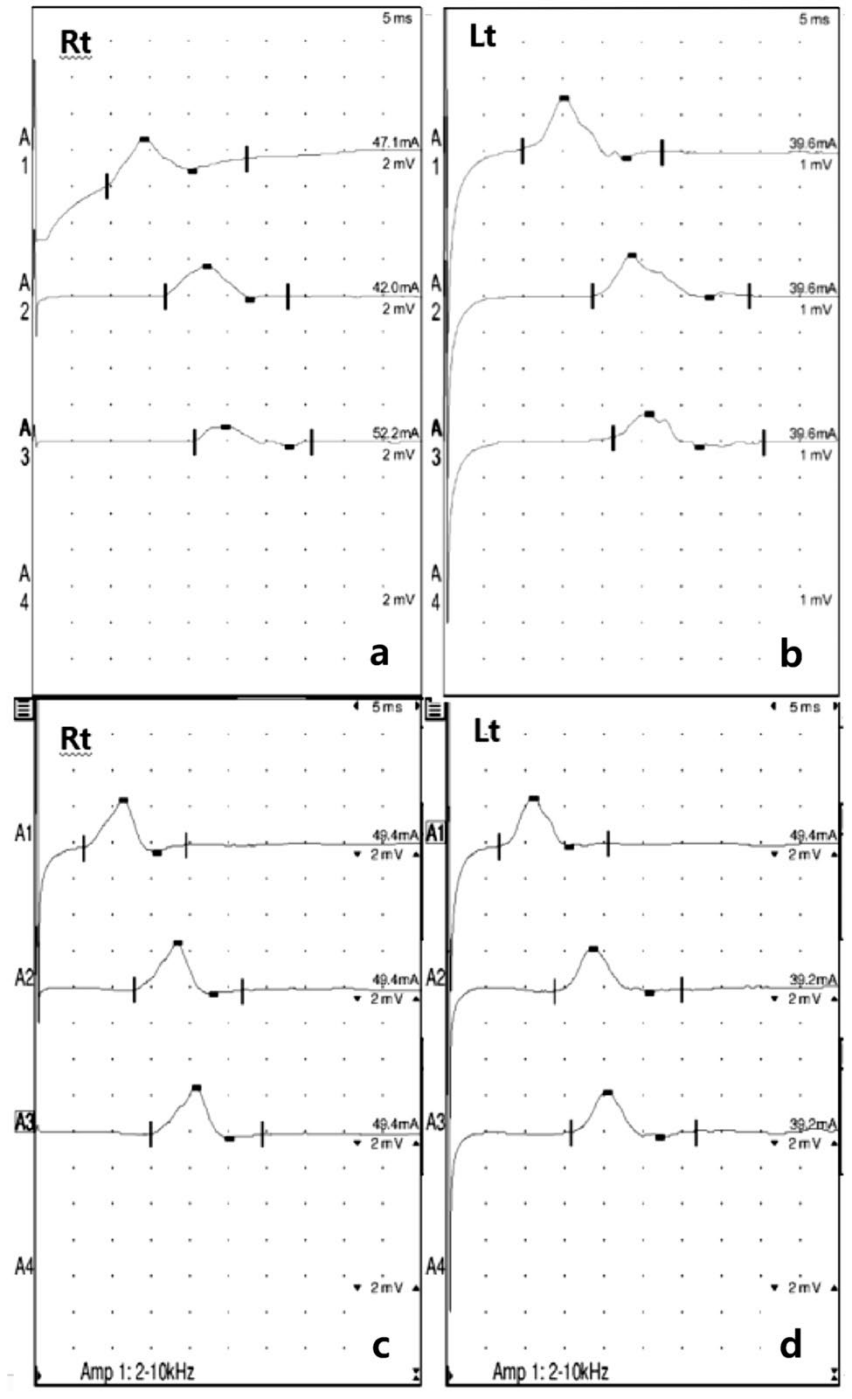
Waveforms of serial nerve conduction studies (NCSs) on bilateral fibular nerves. The results of the first NCS demonstrated demyelinating polyneuropathy presenting with prolonged distal motor latency and conduction failures on bilateral fibular nerves. (**a**) Proximal compound muscle action potential (CMAP) to distal CMAP amplitude ratio of 0.53 in right fibular nerve. (**b**) Proximal CMAP to distal CMAP amplitude ratio of 0.66 in left fibular nerve. In a serial NCS, conduction failures on bilateral fibular nerves disappeared without temporal dispersion by increasing CMAP amplitudes. (**c**) Right fibular nerve. (**d**) Left fibular nerve.

A 50-year-old woman presented with acute progressive upper- and lower-limb weakness. She initially felt clumsiness in both hands 10 days prior and complained of weakness in both ankles and a tingling sensation in the lower extremities 1 week thereafter. Diarrhea was reported as a preceding event that occurred 3 weeks prior. No urination or defecation problems were observed. Neurological examinations revealed bilateral ankle dorsiflexion weakness (Medical Research Council scale grade 3) and decreased deep tendon reflexes on the bilateral ankle. There was no evidence of cranial nerve or sensory system involvement, except for a tingling sensation in the bilateral lower extremities. The first NCS was performed 10 days after onset. The results of the first NCS revealed the presence of demyelinating polyneuropathy presenting with prolonged distal motor latency of the bilateral fibular and tibial nerves with conduction failures (proximal CMAP to distal CMAP amplitude ratios of 0.53 and 0.66 in right and left fibular nerves, respectively), decreased sensory nerve conduction velocity of the median nerve, and prolonged F-wave latencies on the nerves of the upper and lower limbs. The period from disease onset to nadir lasted 11 days; the HFGS at the nadir was 3. The patient was treated with IVIg for five days. The protein level in the CSF analysis was 42 mg/dL, and the result of the anti-ganglioside antibody test was negative. The second NCS was performed 30 days after onset. Improvements in prolonged distal motor latency, sensory nerve amplitude of the median nerve, and F-wave latencies were observed. Conduction failures in the bilateral fibular nerves disappeared without temporal dispersion by increasing CMAP amplitudes (Fig. 2). The HFGS at 1 month from onset was 2, and that at 6 months from onset was 0. The results of the serial NCS are presented in Table 4.

**Table 4:** Serial nerve conduction studies of case patient.

Motor nerve		2015.08			2015.09			Normal values		
		Latency (ms)	Amp (mV)	NCV (m/s)	Latency	Amp	NCV	Latency	Amp	NCV
Median, right	APB-Wr	4.7*	7.7		4.2*	10.8		3.6	> 5.0	
	Wr-Eb		6.4	44*		10.4	50		> 5.0	49.9
	Eb-Ax		6.4	50*		10.4	58		> 5.0	55.9
	F-wave	27.6*			27.0*			26.7		
Ulnar, right	ADM-Wr	3.0*	9.6		2.5	15.0		2.5	> 5.0	
	Wr-Eb		7.4	55		14.4	59		> 5.0	50.6
	Eb-Ax		5.3	67		13.2	64		> 5.0	52.6
	F-wave	28.1*			25.6			26.6		
Tibial,right/left	AH-Ak	10.0*/9.2*	0.5*/1.2*		6.5*/6.3*	5.7/6.8		5.1	> 5.0	
	Ak-P		0.4*/0.7*	32*/38*		5.1/6.1	44/46		> 5.0	40.6
	F-wave	57.0*/56.0*			54.4*/53.0*			46.8		
Fibular, right/left	EDB-Ak	9.4*/9.8*	1.8*/1.7*		6.2*/6.6*	3.0*/2.7*		4.78	> 3.0	
	Ak-P		1.2*/0.9*	24*/35*		2.9*/2.5*	45/42		> 3.0	41.8
	F-wave	60.8*/60.4*			53.5*/53.1*			46.2		

Sensory nerve			Amp (μV)	NCV (m/s)		Amp	NCV		Amp	NCV
Median, right	Wr		11	35*		20	35*		> 10.0	41.2
	Eb		36	53		31	54		> 10.0	49.3
	Ax		22	56		73	65		> 10.0	53.9
Ulnar, right	Wr		10	46		18	47		> 8.0	39.2
	Eb		27	60		59	59		> 10.0	47.4
	Ax		28	75		39	61		> 10.0	48.1
Sural, right/left			23/17	36/35		35/28	43/40		> 6.0	34.6
H reflex, right/left		29.9*/29.3*			28.3/28.1			28.7		

ADM, abductor digiti minimi; AH, abductor halluces; Ak, ankle; Amp, amplitude; APB, abductor pollicis brevis; Ax, axillar; Eb, elbow; EDB, extensor digitorum brevis; NCV, nerve conduction velocity; P, popliteal fossa; Wr, wrist.

*Indicates abnormal values (Reference: Shin J Oh, Clinical electromyography: nerve conduction studies, p86).

## Discussion

In our study, 36 of total 63 patients were diagnosed as AMAN (57.1%) and 27 patients were diagnosed as AIDP (42.9%). RCF was observed in 14 out of 36 (38.9%) in AMAN and 5 out of 27 (18.5%) in AIDP. AIDP patients with anti-ganglioside antibody were 5 out of 17 (29.4%). Onset age of AIDP-RCF was significantly lower than that of AIDP-nRCF and it was similar with AMAN-RCF. Diarrhea was more frequent in AIDP-RCF and AMAN than in AIDP-nRCF as preceding event. HFGSs at 1 month and at 6 months after the onset were significantly lower in AIDP-RCF than in AMAN-nRCF and it was similar with AMAN-RCF. However, there were no significant differences in frequency of anti-ganglioside antibody status among the groups.

GBS subtypes are mainly classified into AIDP and AMAN based on electrodiagnostic studies. In AIDP, demyelination impairs the transmission of impulses along nerve fibers by changing the properties of the paranodal and internodal membranes. As the demyelinating changes take place, the current becomes insufficient to depolarize the node to a threshold, resulting in conduction failure/block, conduction velocity reduction, and temporal dispersion^7^. The pathophysiology of AIDP suggests that the production of autoantibodies by preceding infection and the activity of complement complexes results in the demyelination of peripheral nerves. However, the causative autoantibody for AIDP has not yet been identified^8^. In AMAN, the infecting organisms probably share homologous epitopes with an axon in the node of Ranvier of the peripheral nerves, reflecting molecular mimicry, and the immune responses cross-react with the nerves, causing axonal degeneration^9^. Gangliosides represent major target antigens of axonal GBS. The complement system is activated when IgG antibodies bind to gangliosides located at a node of Ranvier, where GM1 and GD1a are strongly expressed^10^. Axonal degeneration proceeds by forming a membrane attack complex on the axolemma of motor fibers, resulting in the disappearance of voltage-gated sodium channels and the detachment of paranodal myelin loops^11,12^. Anti-ganglioside antibody binding to the nodal axolemma leads to dysfunction of voltage-gated sodium channel clusters and disruption of paranodal axon–Schwann cell adhesion, resulting in conduction failure/blockage. If an autoimmune attack is aborted, axonal degeneration does not progress and nodal function rapidly recovers, which represents the pathomechanism of RCF^12,13^. RCF in AMAN has been considered a sign of nodopathy and has a favorable prognosis^14^. Many previous reports have suggested that AMAN without RCF has the worst prognosis in GBS^15,16^, similar to the results of our study.

The term nodo-paranodopathy was suggested to characterize neuropathies related to autoantibodies based on a common pathogenic mechanism of dysfunction at the node of Ranvier, resulting in a pathophysiological continuum from transitory nerve conduction failure to axonal degeneration^17^. Some common beliefs, such as conduction failure and conduction velocity slowing, are associated only with demyelinating neuropathies and may be misleading in the interpretation of electrophysiological results in disorders affecting the nodal region. Uncini et al.^18^ have suggested that temporal dispersion helps to distinguish conduction failure in demyelinating neuropathy from conduction failure in nodopathy. Remyelination following acute demyelination or ongoing demyelination is characterized by the desynchronization of conduction among fibers, which induces temporal dispersion and decreases CMAP amplitude due to the cancellation effect of opposing phases of a single motor unit potential. On the contrary, conduction failure in nodopathy may be reversed without a temporal dispersion. Conduction velocity slowing is thought to be characteristic of demyelinating neuropathy, but partial inactivation of the sodium channel reduces the conduction velocity in nodopathy and improves in parallel with the resolution of conduction failure^18^.

In our study, conduction failure without temporal dispersion was identified in some patients who were classified as having AIDP in an initial electrodiagnostic study and conduction failure disappeared within a short period of time without temporal dispersion in the serial NCS. We classified these patients as having AIDP with RCF. Temporal dispersion was confirmed in 20 of the remaining 22 patients excluding AIDP-RCF out of total 27 AIDP patients in our study. Although there was no temporal dispersion, delayed terminal latencies and conduction blocks were observed in remaining 2 patients of AIDP. However, because the conduction blocks continued even after 1 month, the 2 remaining patients classified into AIDP-nRCF group. The difference between these two patients and patients with AIDP-RCF group was abnormalities on sensory NCS. All 5 patients in the AIDP-RCF group had no abnormality in sensory NCS like AMAN, but in the 2 remaining patients with AIDP-nRCF, there were abnormalities in sensory NCS. Uncini et al., proposed new criteria based on the results of Hadden^19^ and Rajabally’s criteria^1^, and the new criteria had intermediate features of two previous electrodiagnostic criteria for GBS^20^. In previous study by Uncini et al., RCF in the presence of other demyelinating features occurred in 8/53 patients (15%) with a diagnosis of AIDP, similar to our results. In our study, 5/27 patients (18.5%) were classified into the AIDP-RCF group.

Rajabally et al., suggested modified criteria for classifying patients with GBS. However, these criteria could not confirm reversible conduction failure/block in the initial stage of GBS. In fact, Although approximately 50–65% of patients are initially suggested that they have demyelinating features, some of these cases are often changed into axonal neuropathy with initial conduction failure^21^. The importance of serial NCS for a proper diagnosis is stressed. Uncini et al.^20^ suggested that serial NCS is needed for the accurate classification of GBS subtypes, and temporal dispersion is a main feature of demyelinating GBS. We conducted serial NCS in all enrolled patients and finally made a diagnosis after having checked the results of all serial NCS. In addition, we classified the RCF groups in AMAN and AIDP based on the results of serial NCS.

Diarrhea is often preceded by *Camplyoacter jejuni (C. jejuni)* infection in AMAN, and anti-ganglioside antibodies against GM1 and GD1a are frequently detected. Ogawara et al.^22^ reported that anti-*C. jejuni-*positive patients showed a significantly higher percentage of AMAN than the *C. jejuni-*negative patients (70% of *C. jejuni-*positive patients had AMAN type), and 80% of patients with AMAN had IgG antibodies to GM1, GD1a, GalNAc-GD1a, or GD1b. In our present study, diarrhea was significantly more frequent in AMAN and AIDP with RCF than in AIDP without RCF as a preceding event. In addition, the presence of anti-ganglioside antibody revealed a tendency for higher positivity in AMAN and AIDP with RCF than in AIDP without RCF, but there was no significant difference. Testing for anti-ganglioside antibodies is important because they are related to the pathomechanism of GBS. A recent cohort study from China analyzed the relationship between immune-mediated peripheral neuropathy and serum levels of anti-ganglioside IgG and IgM antibodies^23^. In this cohort, 42.4% patients were positive for at least one of the anti-ganglioside antibodies, and the sensitivity and specificity of the diagnostic test for GBS was 42% and 76%, respectively. The sensitivity of the anti-ganglioside antibody detection method can be elevated if ganglioside complexes are detected rather than each type of ganglioside^3,24^. In our study, we used immunoblotting methods for each type of ganglioside, which might have caused relatively low sensitivity and no statistical differences in anti-ganglioside antibody status among the groups.

Our study had a number of limitations. First, the number of subjects in our study was relatively small, and the study was carried out at a single tertiary center. In particular, the number of patients in the AIDP with RCF group was five, which was insufficient to represent the clinical manifestations of the patients in that group. Further studies that could propose the new additional criteria for classifying GBS is required by collecting and analyzing many cases in various regions. Second, testing for antiganglioside antibodies is critical in terms of the pathomechanism of GBS. However, there were no significant differences in anti-ganglioside antibody status among the groups in our study. More meaningful results can be obtained among the groups if additional tests for *C. jejuni* are conducted.

The current electrodiagnostic criteria for GBS can be used to classify GBS into AIDP, AMAN, and AMAN with RCF but have not suggested a classification of AIDP with RCF. However, some patients had RCF with demyelinating features in the initial NCS and presented different clinical features and prognoses from typical AIDP. A previous review by Yuki reported a study in which *C. jejuni*-related GBS patients were classified as having AMAN (n = 16) or AIDP (n = 5) or were unclassified (n = 1) in the first electrodiagnostic test^25^. Five *C. jejuni*-positive patients with the initial AIDP subtype showed prolonged motor distal latencies in two or more nerves. However, these changes rapidly normalized within 2 weeks. The author suggested that patients with *C. jejuni*-related GBS can show transient slowing of nerve conduction, mimicking demyelination, which is called a pseudo-demyelinating feature in AMAN^25,26^. However, there currently remain no electrodiagnostic criteria for classifying RCF in AIDP.

In conclusion, RCF in AIDP can be observed and is comparable with AMAN with RCF in terms of clinical features. AIDP with RCF may be a manifestation of nodopathy. The current dichotomous electrodiagnostic criteria, classifying demyelinating and axonal neuropathy, are not sufficient to define the presence of nodopathy. Further studies are required to revise the electrodiagnostic criteria for GBS.

## Data Availability

The original contributions presented in the study are included in the article. Further inquiries can be directed to the corresponding author by seh337@daum.net.
